# A novel optical imaging probe for targeted visualization of NLRP3 inflammasomes in a mouse model of age-related macular degeneration

**DOI:** 10.3389/fmed.2022.1047791

**Published:** 2023-01-10

**Authors:** Marcell E. Paguaga, John S. Penn, MD Imam Uddin

**Affiliations:** ^1^Department of Ophthalmology and Visual Sciences, Vanderbilt University School of Medicine, Nashville, TN, United States; ^2^Department of Molecular Physiology and Biophysics, Vanderbilt University School of Medicine, Nashville, TN, United States; ^3^Department of Biomedical Engineering, Vanderbilt University, Nashville, TN, United States

**Keywords:** age-related macular degeneration, NLRP3 inflammasome, macrophages, optical imaging, choroidal neovascularization, CY-09

## Abstract

**Purpose:**

Wet form of age-related macular degeneration (wet AMD) is a progressive vascular disease that mainly affects older adults and causes severe and irreversible vision loss. A key complication of wet AMD is choroidal neovascularization (CNV), which may be driven in part by NLRP3 inflammasomes that are associated with macrophages migration to CNV lesions. Since activated NLRP3 is correlated with CNV, visualizing NLRP3 inflammasomes and their associated macrophages is of great interest to monitor wet AMD progression and develop effective therapies against it. However, to the best of our knowledge, current ophthalmic imaging systems do not permit such targeted imaging. Therefore, in this study, we developed InflammaProbe-1, an optical imaging probe for targeted visualization of NLRP3 inflammasomes in CNV lesions.

**Methods:**

InflammaProbe-1 was synthesized by conjugating a clinically relevant fluorophore, Oregon Green^®^ 488, to the selective NLRP3 inhibitor, CY-09. The ability of InflammaProbe-1 to target NLRP3 was assessed with an enzyme-linked immunosorbent assay by comparing its ability to inhibit NLRP3-mediated secretion of IL-1β to that of CY-09 in LPS-primed and nigericin-stimulated BMDMs. *In vitro* confocal imaging of NLRP3 was performed on InflammaProbe-1-stained BMDMs that had been induced to express NLRP3 with LPS. *In vivo* imaging of NLRP3 was conducted on mouse laser induced choroidal neovascularization (LCNV), a model of AMD, 6 h after an intraperitoneal injection of InflammaProbe-1 at 10 mg/kg on day 4 post-LCNV.

**Results:**

InflammaProbe-1 was just as effective as CY-09 at inhibiting IL-1β secretion (*p* < 0.01 at 10 μM for both the InflammaProbe-1 and CY-09 groups relative to the control). InflammaProbe-1-stained BMDMs that had been induced to express NLRP3 showed significantly brighter fluorescence than untreated cells (*p* < 0.0001 for LPS treatment group and *p* < 0.001 for LPS and nigericin treatment group). Furthermore, *in vivo* molecular imaging of NLRP3 was achieved in mouse LCNV.

**Conclusion:**

We propose that InflammaProbe-1 may be a useful molecular imaging probe to monitor the onset, progression, and therapeutic response of AMD and other NLRP3-mediated diseases.

## Introduction

Age-related macular degeneration (AMD) is a progressive vascular disease that mainly affects adults older than 55 years and causes severe and irreversible visual impairments, accounting for about 6–9% of blindness worldwide (1–3). Early-stage or dry AMD is the most common form, but the most severe form is neovascular or wet AMD—which accounts for 90% of AMD-related blindness (4). While dry AMD is characterized by the formation of drusen in the sub-retinal space, wet AMD is distinguished by the pathological growth of abnormal choroidal blood vessels known as choroidal neovascularization (CNV). The progression of CNV may be critically regulated by vascular endothelial growth factor (VEGF), a protein that stimulates blood vessel growth (5). Predictably, anti-VEGF therapy is highly effective for the management of wet AMD (6). However, many wet AMD patients do not respond favorably to anti-VEGF drugs, (7) which presents a major challenge to clinicians who want to impede the advancement of CNV. Resistance to anti-VEGF treatments may reflect the existence of other important mediators of this disease. Indeed, the pathological progression of wet AMD is driven in part by leukocytes, such as activated monocytes, that migrate to the site of choroidal neovascular lesions where they become macrophages and secrete pro-inflammatory cytokines (8–10). The molecular mechanism of macrophage-mediated inflammation in CNV may involve the NOD-, LRR-, and PYR-containing protein 3 (NLRP3) inflammasome—a multiprotein complex that initiates immune responses after being activated by a variety of stimuli, such as pathogens or cellular damage (11, 12). When NLRP3 is activated, it oligomerizes and binds to an adaptor protein, apoptosis-associated speck-like protein containing a CARD (ASC), and an effector protein, pro-caspase 1. Pro-caspase 1 is then cleaved to its enzymatically active form, caspase 1, which in turn activates pro-inflammatory cytokines, such as interleukin-1β (IL-1β), that are secreted by the cell (13). Expectedly, a study showed that IL-1β levels were increased 4-fold in the vitreous of patients with wet AMD in comparison to the control group (14).

Since NLRP3 is associated with CNV, (15) visualizing NLRP3 inflammasomes and their associated macrophages is of great interest to clinicians and researchers who aim to monitor and study the progression of AMD. In turn, this may enable the development of effective therapies for patients who do not respond to anti-VEGF drugs. However, to the best of our knowledge, existing ophthalmic imaging systems do not permit targeted imaging of NLRP3 in activated macrophages. For instance, Optical Coherence Tomography (OCT) is a non-invasive and high resolution imaging technology that is widely used to diagnose AMD, but it cannot effectively distinguish immune cells from retinal pigmented epithelial cells or other cells (10). A related imaging modality, Adaptive Optics Scanning Laser Ophthalmoscopy (AO-SLO), was recently used to study the spatiotemporal dynamics of GFP^+^ microglia in a mouse model of photoreceptor damage, (16) but this method does not provide key information about the presence or absence of NLRP3 inflammasomes in microglia.

Due to the association between NLRP3 inflammasomes, activated macrophages and CNV, (15) we hypothesized that an NLRP3-targeted fluorescent probe would enable visualization of pro-inflammatory macrophages in CNV. Recently, a group of researchers synthesized an NLRP3-targeted fluorescent probe by conjugating a fluorophore, coumarin 343, to the NLRP3 inhibitor, MCC950 (17). Although this is a promising new probe that enabled visualization of NLRP3 in cells, it is not suitable for ophthalmic *in vivo* applications. In the current study, we developed an NLRP3-targeted optical imaging probe, InflammaProbe-1, by conjugating an optimal fluorophore, Oregon Green^®^ 488, to the selective NLRP3 inhibitor, CY-09 (18). In addition, we demonstrated applications of this novel technology for *in vivo* imaging of NLRP3 inflammasomes and their associated macrophages in a mouse model of wet AMD. Herein we present our results.

## Results

### Design and synthesis of InflammaProbe-1

**FIGURE 1:**
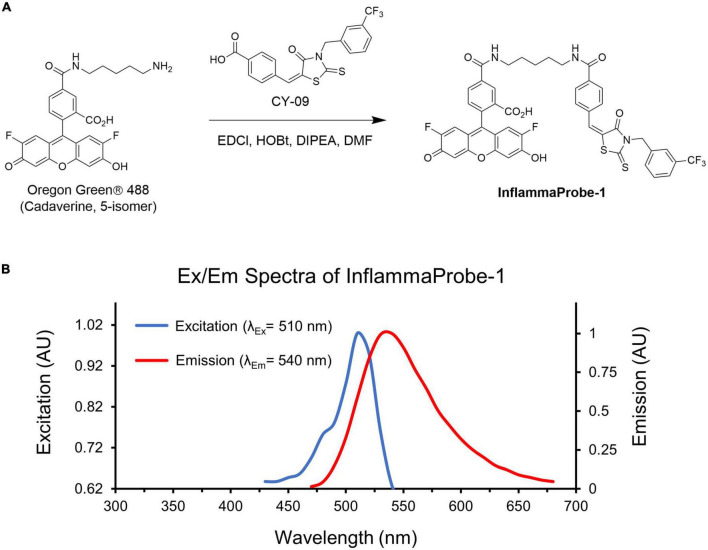
Synthesis and characterization of InflammaProbe-1. **(A)** InflammaProbe-1 was synthesized by conjugating a selective inhibitor of NLRP3 (CY09) to a commercially available fluorophore (Oregon Green^®^ 488). Conjugation was achieved with an EDCI-mediated coupling method. **(B)** Excitation and emission spectra of InflammaProbe-1 in ethanol containing 10% DMSO.

We designed our NLRP3-targeted optical imaging probe, InflammaProbe-1, by conjugating two components: (1) A fluorophore that would allow fluorescence-based visualization, and (2) a selective NLRP3 inhibitor that would enable targeting of NLRP3. With respect to the fluorophore, we chose Oregon Green^®^ 488, a bright and widely used dye derivatized from the FDA-approved fluorescein. Indeed, fluorescein angiography (FA) is a common imaging method used in most ophthalmic clinics. For the NLRP3 inhibitor, we selected CY-09, a molecule that has been shown to inhibit NLRP3 selectively and directly by binding to its NACHT domain, thereby blocking activation of the NLRP3 inflammasome. When tested in a mouse model, CY-09 suppressed NLRP3-mediated cellular secretion of IL-1β and alleviated inflammatory disorders (18). Therefore, in our current study, we synthesized InflammaProbe-1 by conjugating the selective inhibitor of NLRP3, CY-09, to the fluorophore, Oregon Green^®^ 488 (Figure 1A). We observed a slight redshift of its excitation and emission maxima (λ_ex_/λ_em_ = 510/540 nm) relative to those of Oregon Green^®^ 488 (λ_ex_/λ_em_ = 490/520 nm), possibly due to conjugation with CY-09 (Figure 1B). Spectroscopic analyses of InflammaProbe-1, including high resolution mass spectrometry (HRMS) and proton nuclear magnetic resonance (^1^H-NMR), were consistent with its predicted mass and structure, respectively (Supplementary Figure 2 and Supplementary material).

### InflammaProbe-1 targets the NLRP3 inflammasome

**FIGURE 2:**
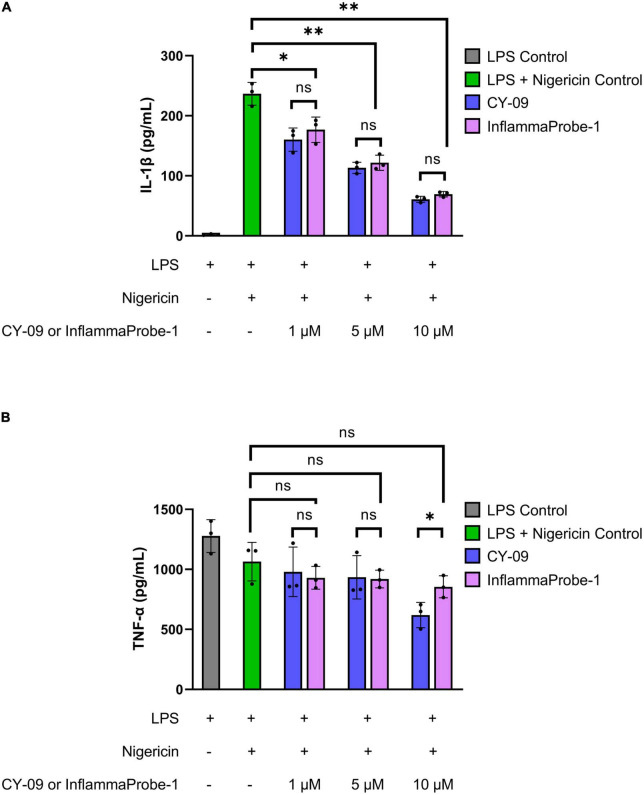
InflammaProbe-1 inhibits NLRP3-mediated secretion of IL-1β but not TNF-α. **(A)** InflammaProbe-1 and CY-09 dose-dependently inhibited NLRP3-mediated secretion of IL-1β in LPS-primed and nigericin-stimulated mouse bone marrow-derived macrophages. **(B)** InflammaProbe-1 and CY-09 had no significant effect on LPS-induced secretion of TNF-α in comparison to the LPS and nigericin control, except for CY-09 at 10 μM. These data suggest that InflammaProbe-1 retains the inhibitory ability of its parent compound, CY-09, that enables it to target the NLRP3 inflammasome. Levels of IL-1β and TNF-α were measured by performing ELISA. The data were expressed as the mean ± SD (*n* = 3). Statistical analysis by unpaired *t*-tests with Welch’s corrections; *p < 0.05 and **p < 0.01.

Following synthesis and characterization, we confirmed InflamamProbe-1’s ability to target NLRP3 by comparing its inhibitory ability to that of its parent compound, CY-09, using enzyme-linked immunosorbent assay (ELISA). As shown in Figure 2A, both CY-09 and InflammaProbe-1 dose-dependently inhibited NLRP3-mediated secretion of IL-1β in LPS-primed and nigericin-stimulated mouse bone marrow-derived macrophages (BMDM). InflammaProbe-1 was just as effective as CY-09 at inhibiting NLRP at all three concentrations that were tested. At 1, 5, and 10 μM, both compounds caused a ∼1.3-fold decrease (*p* < 0.05), ∼2-fold decrease (*p* < 0.01), and ∼4-fold decrease (*p* < 0.01), respectively, of IL-1β levels in comparison to the LPS and nigericin control. To test for an undesired off-target effect, the cell supernatants were also assayed for tumor necrosis factor-α (TNF-α). As expected, InflammaProbe-1 had no significant effect on LPS-induced secretion of TNF-α in comparison to the LPS and nigericin control (Figure 2B). CY-09 showed similar results at 1 and 5 μM, but not at 10 μM; at this concentration, CY-09 significantly decreased the levels of TNF-α in comparison to InflammaProbe-1 (*p* < 0.05). Overall, these results suggest that InflammaProbe-1 retains the inhibitory properties of CY-09 that enable it to target the NLRP3 inflammasome.

### *In vitro* imaging of NLRP3 in activated BMDMs

**FIGURE 3:**
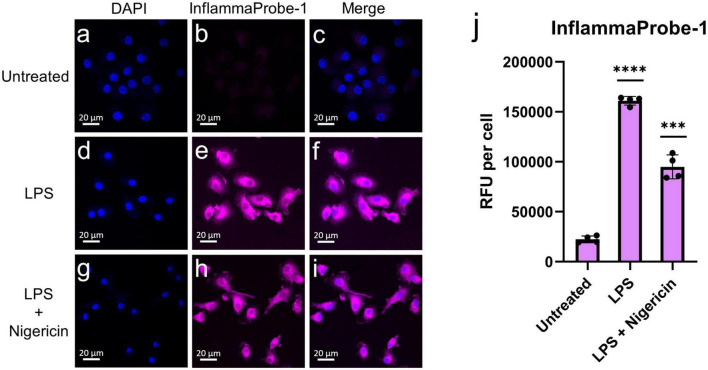
*In vitro* imaging of NLRP3 in bone marrow-derived macrophages (BMDMs) using InflammaProbe-1. **(a–c)** Untreated BMDMs, **(d–f)** LPS-primed BMDMs, and **(g–i)** LPS-primed and nigericin-stimulated BMDMs that were treated with 10 μM InflammaProbe-1, fixed on microscope slides, and imaged using confocal fluorescence microscopy. **(j)** Quantification of InflammaProbe-1 RFUs per cell of confocal microscopy images. Relative to the untreated control cells, those that were treated with LPS alone or with LPS and nigericin displayed significantly brighter InflammaProbe-1-dependent fluorescence (*p* < 0.0001 and *p* < 0.001, respectively). These results indicate that InflammaProbe-1 selectively stains macrophages that have been induced to express NLRP3. These data are representative of four replicates from each experimental group. Statistical analysis by unpaired *t*-tests with Welch’s corrections; ***p < 0.001 and ****p < 0.0001 relative to the untreated control.

After confirming InflammaProbe-1’s ability to target NLRP3, we used it to visualize NLRP3 in activated macrophages *in vitro*. Three different groups of BMDMs—untreated BMDMs, LPS-primed BMDMs, and LPS-primed and nigericin-stimulated BMDMs—were stained with InflammaProbe-1, fixed on microscope slides, and imaged using confocal fluorescence microscopy. As expected, untreated BMDMs showed minimal InflammaProbe-1-dependent fluorescence (Figures 3a–c). In contrast, LPS-primed BMDMs displayed significant fluorescence primarily in the cytosol (*p* < 0.0001; Figures 3d–f, j). Since NLRP3 localized to endoplasmic reticulum structures in the cytosol and after activation NLRP3 redistribute to the perinuclear space (19), we tested InflammaProbe-1 for imaging activated NLRP3 in LPS-primed and nigericin-stimulated BMDMs. Significant fluorescence with punctate-pattern fluorescence were observed in LPS-primed and nigericin-stimulated BMDMs (*p* < 0.001; Figures 3g–j). We observed decreased fluorescence per cell in LPS-primed and nigericin-stimulated BMDMs compared to LPS alone treated BMDMs, may be due to reduced expression of NLRP3 in LPS-primed and nigericin-stimulated BMDMs. Though, CY09 and InflammaProbe-1 could inhibit NLRP3 oligomerization and inflammasome assembly as shown in Figure 2A, however both compounds may not have any effect on NLRP3 expression (18). These results indicate that InflammaProbe-1 could detect macrophages that are associated with NLRP3 inflammasomes.

### *In vivo* imaging of NLRP3 in LCNV

**FIGURE 4:**
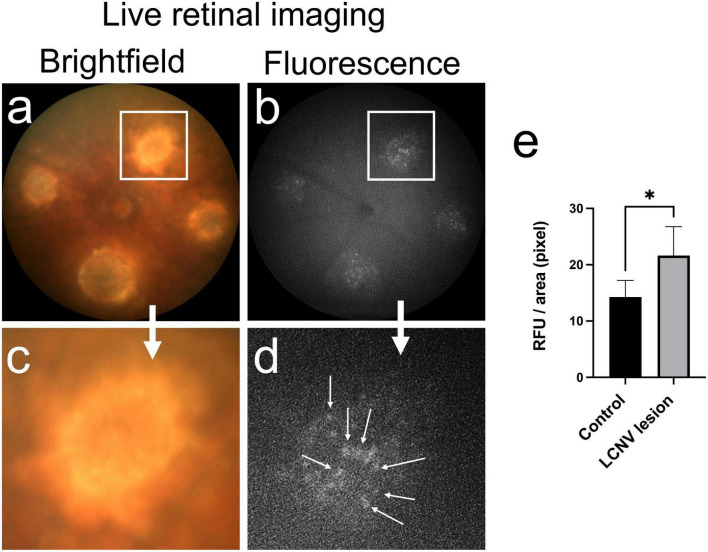
*In vivo* retinal imaging of NLRP3 in laser-induced choroidal neovascularization (LCNV) using InflammaProbe-1. **(a)** Brightfield and **(b)** fluorescence fundus images of mouse LCNV taken 6 h after a 10 mg/kg intraperitoneal injection of InflammaProbe-1 on day 4 post-LCNV. The fluorescence fundus image shows InflammaProbe-1-dependent fluorescence that is localized to each of the LCNV lesions observed in the brightfield fundus image. These are representative images of 12 mouse LCNV eyes. **(c,d)** Higher magnification of panels **(a,b)**, respectively. **(e)** Fluorescence intensity measurements expressed in relative fluorescence units (RFU) per pixel area were measured using ImageJ software. Statistically significant fluorescence enhancement was observed in LCNV lesions compared to those in the non-laser control groups. Statistical significance *p < 0.05.

Next, we used InflammaProbe-1 to visualize NLRP3 inflammasomes in mouse laser-induced choroidal neovascularization (LCNV), a well-established model of wet AMD (20, 21). Four days post-induction of LCNV, inflammaProbe-1 was injected intraperitoneally into mice. Six hours post-injection, brightfield and fluorescence fundus images showed cellular localization of InflammaProbe-1-dependent fluorescence to each of the four CNV lesions (Figure 4). Notably, the density of InflammaProbe-1-positive cells was higher at the center of the lesions than their periphery. As expected, there was minimal background fluorescence in the non-lesioned regions, which served as the healthy controls. Additionally, no detectable fluorescence was observed in the LCNV lesions of mice that had been injected with an equimolar dose of Oregon Green dye control (Supplementary Figure 3). To the best of our knowledge, this is the first evidence that NLRP3 inflammasomes and their associated activated cells can be visualized in a living ocular disease model.

### *Ex vivo* imaging of NLRP3 in LCNV

**FIGURE 5:**
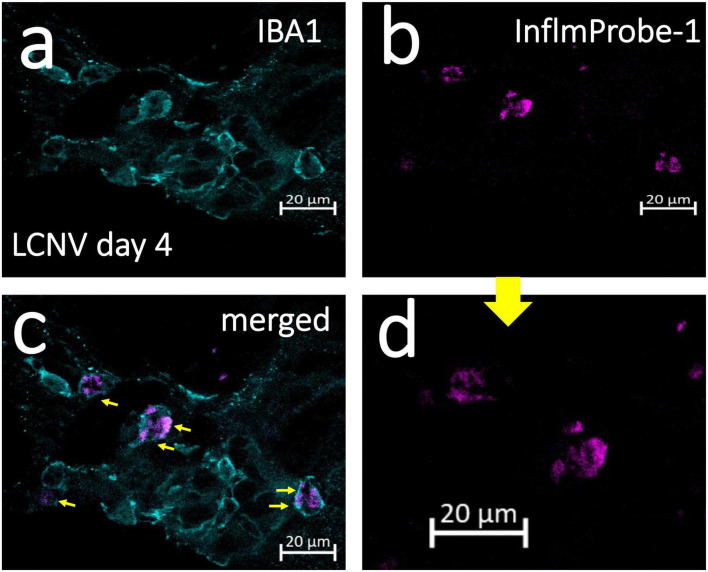
Confocal imaging of NLRP3 in laser induced choroidal neovascularization (LCNV) tissues using InflammaProbe-1. Four days after LCNV, mice were intraperitoneally injected with InflammaProbe-1 at 10 mg/kg and enucleated after 6 h. Their choroids were dissected and co-stained with fluorescently tagged antibodies against IBA1, which targets macrophages. The stained choroidal lesions were then imaged with confocal fluorescence microscopy at 63x magnification **(a–c)**. **(d)** Higher magnification of panel **(b)**. The yellow arrows in panel **(c)** indicate InflammaProbe-1 localization in macrophages. These are representative confocal images taken from 12 choroids. See also Supplementary Figure 4 for co-localization of InflammaProbe-1 in 3D reconstructed LCNV lesions from confocal images.

Following *in vivo* imaging of NLRP3 in mouse LCNV, we investigated InflammaProbe-1’s specificity for macrophages by comparing the co-localization of InflammaProbe-1, macrophages, and another prevalent cell type at the choroidal neovascular lesion—endothelial cells. To do so, the dissected choroids were co-stained with fluorescently tagged antibodies against ionized calcium binding adaptor molecule 1 (IBA1)—a selective marker for microglia/macrophages (22)—and Isolectin B4 (IB4)—a marker that stains endothelial cells, (23) but which has also been shown to stain macrophages (24–26). Confocal fluorescence imaging of the choroidal tissues revealed substantial co-localization of InflamamProbe-1 with IBA1^+^ macrophages and microglial cells (Figures 5a–d). InflammaProbe-1 and its associated macrophages and microglial cells were dispersed throughout the lesion, including the center and periphery. Since InflammaProbe-1 is specific for NLRP3 inflammasomes, NLRP3 positive microglial cells beside choroidal areas could also be detected using InflammaProbe-1. A quantitative correlation analysis was performed using three-dimensional reconstructed CNV lesions to co-localize InflammaProbe-1-associasted fluorescence with macrophages and endothelial cells (Supplementary Figure 4) (27). There was a high degree of correlation between InflammaProbe-1 and IBA1, which stains macrophages (*r* = 0.81; Supplementary Figure 4C). The reduced correlation was observed in InflammaProbe-1 and IB4, which stains primarily endothelial cells (*r* = 0.48; Supplementary Figure 4B); although an *r* value of 0.48 still represents a moderate degree of correlation, may be due to non-specific staining of macrophages by IB4. This explanation is consistent with the high degree of correlation found between IBA1 and IB4 (*r* = 0.66; Supplementary Figure 4A) although it was still lower than the correlation between IBA1 and InflammaProbe (*r* = 0.81). Overall, these data suggest that InflammaProbe-1 targets NLRP3-associated macrophages. In addition, we confirmed that NLRP3 immuno-fluorescence was co-localized in LCNV lesion as shown in Supplementary Figure 5, suggesting that NLRP3 inflammasomes are associated with CNV lesions.

### Toxicity of InflammaProbe-1

**FIGURE 6:**
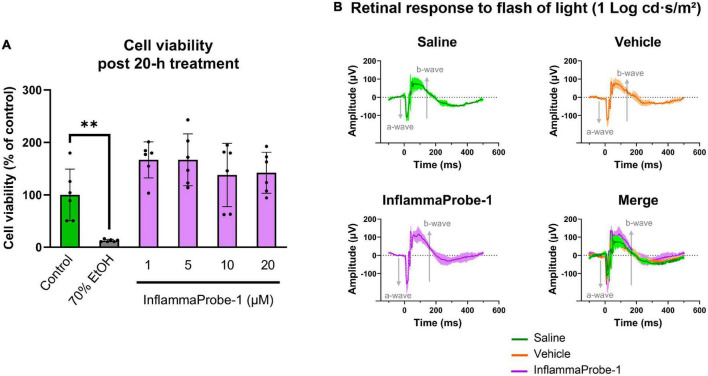
Toxicity of InflammaProbe-1. **(A)** Cytotoxicity of InflammaProbe-1 was assessed in primary mouse retinal microvascular endothelial cells (MRMEC) using a fluorescence-based assay with Calcein Deep Red™ AM ester. The viability of MRMECs was not significantly reduced by a 20-h exposure to 1–20 μM InflammaProbe-1 in comparison to untreated cells. **(B)** Retinal toxicity was assessed in dark-adapted mice using ganzfeld electroretinography (ERG) 7 days after an intraperitoneal injection of InflammaProbe-1 at 10 mg/kg. Relative to the retinas of mice injected with saline or a vehicle control (10% DMSO in PBS), the retinas of mice that had been injected with InflammaProbe-1 did not show any significant reduction in the a-wave and b-wave amplitudes of their electrical response to a 1 Log cd⋅s/m^2^ light flash. These results suggest that InflammaProbe-1 is not toxic to primary cells or retinal tissues. Cell viability data were expressed as the mean ± SD of six replicates from each group. Statistical analysis by unpaired *t*-tests with Welch’s corrections; **p < 0.01. ERG data were expressed as the mean ± SD of four retinas from each group.

Finally, we assessed the toxicity of InflammaProbe-1 on primary cells and retinal tissues using *in vitro* and *in vivo* assays. To assess the probe’s cytotoxicity *in vitro*, we performed a Calcein Deep Red AM*™* assay on primary mouse retinal microvascular endothelial cells (MRMECs). As shown in Figure 6A, a 20-h exposure of InflammaProbe-1 at up to 20 μM did not significantly reduce the viability of MRMECs in comparison to the untreated control group. In contrast, the positive control of 70% ethanol significantly decreased cell viability (*p* < 0.01). Next, we assessed *in vivo* retinal toxicity in mice 7 days after an IP injection of InflammaProbe-1. Using dark-adapted, ganzfeld electroretinography (ERG), we stimulated the mice’s retinas with flashes of light and evaluated their electrical response. Specifically, we analyzed the amplitude of the initial hyperpolarizing a-wave—which originates from photoreceptors—and the subsequent depolarizing b-wave—which is produced by cells that are post-synaptic to photoreceptors, including muller cells and on-bipolar cells (28, 29). As shown in Figure 6B, the retinas of mice injected with InflammaProbe-1 did not show any significant reduction in the a-wave and b-wave amplitudes relative to the retinas of mice injected with saline or a vehicle control. Based on these assays, InflammaProbe-1 does not show any sign of toxicity to primary cells or retinal tissues.

## Discussion

Though several methods have been developed to detect inflammasome activation by imaging caspase-1 and other mediators activity *in vitro* and *in vivo*, applications to detect NLRP3 inflammasomes in retinal diseases have been limited (30). In this study, we hypothesized that we could develop an NLRP3-targeted optical imaging probe that would enable us to image NLRP3 inflammasomes in activated macrophages in living ocular tissues. To do so, we first synthesized InflammaProbe-1 by conjugating the fluorophore, Oregon Green^®^ 488, to the selective NLRP3 inhibitor, CY-09. Next, we confirmed its ability to target NLRP3 and used it to visualize NLRP3 in LPS-primed and nigericin-stimulated mouse macrophages. Then, using InflammaProbe-1, we performed *in vivo* imaging of NLRP3 inflammasomes in LCNV, a mouse model of wet AMD. To the best of our knowledge, this is the first evidence of *in vivo* molecular imaging of NLRP3 inflammasomes achieved in an ocular disease model. Subsequent *ex vivo* imaging of stained choroidal neovascular lesions confirmed substantial co-localization of InflammaProbe-1 and macrophages. Finally, InflammaProbe-1 did not appear to be toxic to primary cells or retinal tissues, as indicated by *in vitro* and *in vivo* cytotoxicity assays.

It should be noted that InflammaProbe-1 displayed slightly red shifted ex/em maxima relative to its parent fluorophore, Oregon Green^®^ 488, which is why we used a laser set to 510 nm to achieve optimal *in vitro* and *ex vivo* visualization of NLRP3 inflammasomes. Nonetheless, we were still able to use a 488 nm laser to capture high quality *in vivo* images of individual NLRP3-associated cells that had localized to CNV lesions.

We reiterate that InflammaProbe-1 enabled us to conduct *in vivo* imaging of NLRP3—an accomplishment that had not been previously reported in the literature. Based on our findings and on the reported association between inflammasomes and wet AMD, we propose that our newly developed optical imaging technology may be a useful tool to study the onset, progression, and therapeutic response of wet AMD. It may also help answer questions about relevant molecular and cellular mechanisms involved in this vascular disease. For instance, the specific role of macrophages in the pathological progression of wet AMD remains largely unknown. Additionally, the mechanism by which monocyte-derived macrophages are recruited from peripheral circulation to the site of CNV is not clear. Elucidating such mechanisms may in turn help develop effective therapies for the large percentage of wet AMD patients who do not respond to anti-VEGF drugs.

Finally, we emphasize that the utility of InflammaProbe-1 is not limited to AMD; its applications may be extended to other inflammatory diseases, such as proliferative diabetic retinopathy (31, 32), that are mediated by the NLRP3 inflammasome.

## Materials and methods

### Synthesis of InflammaProbe-1

InflammaProbe-1 was synthesized by adding CY-09 (11.8 μmol), 1-hydroxybenzotriazole hydrate (HOBt; 11.8 μmol), N, N- diisopropylethylamine (DIPEA; 11.8 μmol), and 1-(3-dimethylaminopropyl)-3-ethylcarbodiimide hydrochloride (EDCl; 11.8 μmol) to a stirred solution of Oregon Green^®^ 488 Cadaverine, 5-isomer (10.07 μmol) in dimethyl formamide (DMF; 2 ml) at 25°C. The resultant mixture was stirred for 2 days at 25°C. Removal of the solvent *in vacuo* afforded a residue that was purified by silica gel column chromatography using CHCl_3_:MeOH:NH_4_OH (35:7:1) to give an orange solid. Yield = 31%. InflammaProbe-1 was dissolved in DMSO to a 1 mM stock solution for experimental procedures, unless otherwise indicated. The synthesis scheme shown in Figure 1B was created with ChemDraw Professional (V20.1.1; PerkinElmer Informatics, Inc., Waltham, MA, USA).

### Characterization of InflammaProbe-1

Excitation and emission spectral scan (in EtOH containing 10% DMSO), λ_Ex_/λ_Em_ = 510/540 nm (Figure 1B). HPLC (λ_Ex_ = 510 nm), *t*_*R*_ = 14.15 min, 95% purity (Supplementary Figure 1). HRMS (ESI+): m/z [M + H]^+^ calcd for C_45_H_32_F_5_N_3_O_8_S_2_, 902.1624; Found, 902.1647 (Supplementary Figure 2). ^1^H-NMR (400 MHz, MeOD): δ 8.79 (br,1H), 8.45 (br,1H), 8.22-8.17 (m,1H), 8.08 (m, 2H), 7.95 (m, 1H), 7.78 (m, 2H), 7.69 (m, 2H), 7.61 (m, 2H), 7.58 (m, 1H), 7.48 (m, 1H), 7.40 (m, 1H), 7.36 (m, 1H), 6.82 (d, J = 7.6 Hz, 1H), 6.39 (d, J = 11.2 Hz, 1H), 5.43 (s, 2H), 3.52 (m, 4H), 1.76 (m, 4H), 1.56 (m, 2H).

### Animals

C57BL/6 mice, 4–6 weeks of age, were obtained from Charles River Laboratories (Wilmington, MA, USA). On average, mice weighed 22 g. Refer to Supplementary material for details regarding animal feeding, housing, and sacrifice. All animal procedures were approved by the Vanderbilt University Institutional Animal Care and Use Committee and were in accordance with the ARVO Statement for the Use of Animals in Ophthalmic and Vision Research and in compliance with ARRIVE guidelines.

### Cell culture

Bone marrow-derived macrophages (BMDM), isolated from adult C57BL/6 mouse bone marrow, were purchased from ScienCell Research Laboratories (Carlsbad, CA, USA) and cultured in ScienCell’s phenol red-free Macrophage Medium (MaM) supplemented with 5% fetal bovine serum (FBS), Macrophage Growth Supplement (MaGS), and 1% Penicillin-Streptomycin (Pen, 100 U/ml; Strep, 100 μg/ml). Mouse Primary Retinal Microvascular Endothelial Cells (MRMEC), isolated from C57BL/6 mice, were purchased from Cell Biologics Inc. (Chicago, IL, USA) and cultured in Cell Biologics’ phenol red-free Endothelial Cell Medium supplemented with 0.1% VEGF, 0.1% Heparin, 0.1% EGF, 0.1% ECGS, 0.1% Hydrocortisone, 2 mM L-Glutamine, and 1% Antibiotic-Antimycotic. The medium was additionally supplemented with 10% FBS (R&D Systems, Minneapolis, MN, USA). BMDMs and MRMECs were incubated at 37°C, 5% CO_2_, and 95% relative humidity.

### Inhibition assay of IL-1β and TNF-α

Bone marrow-derived macrophages were seeded in 12-well plates at a density of 5 × 10^4^ cells per well. After an overnight incubation, the cells were primed with 50 ng/ml LPS for 3 h, treated with 1 to 10 μM InflammaProbe-1 or CY-09 for 1 h, and stimulated with 10 μM nigericin for another hour to induce NLRP3 activation, as described in the literature (18, 33). Cell culture supernatants were assayed for mouse IL-1β and TNF-α by performing enzyme-linked immunosorbent assays (ELISA; Invitrogen, Waltham, MA, USA) in accordance with the manufacturer’s instructions. The data were expressed as the mean ± SD of three replicates per group.

### *In vitro* imaging of NLRP3 in BMDMs

Bone marrow-derived macrophages were seeded in 4-chamber slides (Thermo Fisher Scientific, Waltham, MA, USA) at a density of 1 × 10^5^ cells per chamber. After an overnight incubation, the cells were primed with 50 ng/ml LPS for 3 h, treated with 10 μM InflammaProbe-1 for 1 h, and stimulated with 10 μM nigericin for another hour to induce NLRP3 activation, as described in the literature (18, 33). Then, the cells were washed twice with PBS, fixed with 4% neutral buffered formalin (NBF) for about 2 min, and washed again twice with PBS. Immediately, the chambers were removed from the microscope slides in accordance with the manufacturer’s instructions. Cells were mounted with Prolong™ Diamond Antifade Mountant with DAPI (Invitrogen, Waltham, MA, USA) and imaged through confocal fluorescence microscopy using an LSM 710 inverted microscope (Zeiss™, Jena, Germany). The InflammaProbe-1 fluorescence intensity of each image was expressed as RFU per cell, which was calculated by dividing the raw integrated density, measured with Fiji ImageJ2 software, by the number of DAPI-stained nuclei present in the image. The data were representative of four replicates per group.

### *In vivo* imaging of NLRP3 in LCNV

Induction of laser-induced choroidal neovascularization (LCNV) was performed in six adult C57BL/6 mice, three of each sex, following published protocols (20, 21, 34). Briefly, following anesthetization and pupillary dilation, four laser-induced choroidal lesions were created in each eye by rupturing the Bruch’s membrane with a solid-state laser photocoagulator, GYC-500 (Green photocoagulation 532 nm laser) mounted on a slit-lamp (Nidek, Fremont, CA, USA). Lesions were created using the following laser parameters: 100 μm spot size, 0.1 s duration, and 0.1 Watts. Rupture of the Bruch’s membrane was confirmed using OCT imaging (Phoenix Research Laboratories, Pleasanton, CA, USA) of the mouse LCNV as shown in Supplementary Figure 6. Four days later, the LCNV mice were injected intraperitoneally with 10 mg/kg InflammaProbe-1 or an equimolar dose of Oregon Green 488 cadaverine, 5-isomer (5.46 mg/kg) in 100 μL PBS with 10% DMSO. Brightfield and fluorescent fundus images were acquired 6 h post-injection using the Micron IV retinal imaging system (Phoenix Research Laboratories, Pleasanton, CA, USA). Annotations were added to both images using PowerPoint (Microsoft, Redmond, WA, USA). The images were representative of 12 eyes.

### *Ex vivo* imaging of NLRP3 in LCNV

After *in vivo* imaging, the LCNV mice that had been IP injected with InflammaProbe-1 at 10 mg/kg were sacrificed and enucleated. Then, the choroids were dissected and co-stained with DyLight^®^ 649-conjugated IB4 and Alexa Fluor^®^ 594-conjugated antibody against anti-IBA1. See Supplementary material for more details on the immunostaining procedure. The stained tissues were mounted on microscope slides with Prolong™ Diamond Antifade Mountant with DAPI (Invitrogen, Waltham, MA, USA) and imaged through confocal fluorescence microscopy using an LSM 710 inverted microscope (Zeiss™, Jena, Germany). The images were representative of 12 choroids.

### 3-D reconstruction of stained LCNV lesion and correlation analysis

A 33 μm thick Z-stack of a stained LCNV lesion was captured at 63x magnification using confocal fluorescence microscopy. Then, the Z-stack was used to construct a 3-D model and calculate Pearson’s correlation coefficients (*r*) with Imaris software (V9.8.0; Oxford Instruments, Abingdon, UK).

### Cell viability assay

MRMECs (Passage 4) were seeded on sterile black 96-well plates in complete medium at a density of 1.5 × 10^4^ cells per well. When the cells reached 80% confluence, they were treated with 1 to 20 μM InflammaProbe-1 in complete medium or 70% ethanol in water as the positive control for 20 h. The cells that were treated with ethanol were seeded on an identical but separate plate to prevent ethanol vapor from affecting the other experimental groups. After treatment, the cells were washed with HBSS containing Ca^2+^ and Mg^2+^. Then, to assay cell viability, the cells were exposed to an HBSS solution containing 5 μM Calcein Deep Red*™* AM ester (AAT Bioquest, Sunnyvale, CA, USA), a non-fluorescent compound that is cleaved into a brightly fluorescent product (λ_*ex*_/λ_*em*_ = 643/663 nm) by esterases within live cells. This fluorescent compound is red shifted relative to InflammaProbe-1 (λ_*ex*_/λ_*em*_ = 510/540 nm), which was necessary to avoid interference from any InflammaProbe-1-dependent fluorescence during fluorometric measurements. The cells were incubated at 37°C for 1 h. Finally, fluorometric measurements were performed at λ_*ex*_/λ_*em*_ = 620/660 nm using the Cytation 5 microplate reader (BioTek Instruments, Inc., Winooski, VT, USA). Fluorescence intensities were plotted as the percentage of cell viability relative to the control group. The data were expressed as the mean ± SD of six replicates per group.

### Electroretinography

Healthy, adult C57BL/6 mice were injected intraperitoneally with InflammaProbe-1 at 10 mg/kg in 100 μL PBS with 10% DMSO, 100 μL PBS with 10% DMSO as the vehicle control, or 100 μL 0.9% saline as an additional control. Six days post-injection, the mice were dark-adapted inside a ventilated box overnight. After dark-adaptation, *in vivo* retinal toxicity was assayed through electroretinography (ERG) under dim red light in accordance with published methods (34–36). Briefly, after anesthetization and pupillary dilation, the retinas were stimulated with flashes of white light (6,500 K) ranging from −4 to 2 Log cd⋅s/m^2^ using the ganzfeld ColorDome*™* (E3 Research ERG system from Diagnosys LLC, Lowell, MA, USA). Electrical responses were recorded using Espion software (V6, Diagnosys LLC, Lowell, MA, USA) and plotted as voltage amplitude over time. Data were expressed as the mean ± SD of four retinas per group. Refer to Supplementary material for additional details regarding ERG setup.

### Statistical analysis

All experiments were performed at least three biological replicates with similar results and the representative data from a single experiment represents three technical replicates as data presented in this manuscript. Data were expressed as mean ± SD. Statistically significant differences between groups were determined by conducting unpaired *t*-tests with Welch’s corrections. Welch’s *t*-test is an adaptation of student’s *t*-test and is more reliable for statistical analysis to compare unpaired samples. Statistical significance was defined as *p* ≤ 0.05 (*), *p* ≤ 0.01 (**), *p* ≤ 0.001 (***), and *p* ≤ 0.0001 (****). Statistical analyses and graphing were performed using Prism software (V9.3.0, GraphPad, San Diego, CA, USA).

## Data availability statement

The original contributions presented in this study are included in the article/Supplementary material, further inquiries can be directed to the corresponding author.

## Ethics statement

This animal study was reviewed and approved by Vanderbilt University Institutional Animal Care and Use Committee.

## Author contributions

MP designed and performed the experiments, collected and analyzed the data, and wrote the manuscript. JP helped to revise the manuscript. MU conceived and supervised the project, designed, and synthesized compounds, designed and performed experiments, and revised the manuscript. All authors contributed to the article and approved the submitted version.

## Supplementary material

The following should appear as Supplementary material: Supplementary methods including additional details pertaining to reagents, chemical synthesis and characterization, animal feeding and housing conditions, immunostaining procedure, animal anesthesia, and ERG setup; Supplementary Figures including HPLC chromatogram (Supplementary Figure 1), HRMS spectrum (Supplementary Figure 2), *in vivo* retinal images of Oregon Green^®^ 488 dye control (Supplementary Figure 3), and degree of co-localization of InflammaProbe-1 fluorescence with IBA1 and IB4 in LCNV lesion (Supplementary Figure 4); co-localization of NLRP3 in LCNV lesion (Supplementary Figure 5) and Supplementary Tables containing information regarding microscope configurations and image processing steps (Supplementary Tables 1–4).
