# Supplementary material for: A novel optical imaging probe for targeted visualization of NLRP3 inflammasomes in a mouse model of age-related macular degeneration
Source: Front Med (Lausanne). 2023 Jan 10;9:1047791. doi: 10.3389/fmed.2022.1047791 (PMC9871584; doi:10.3389/fmed.2022.1047791)
Supplement: Supplementary file 1 [file Data_Sheet_1.PDF]

Supplementary Material

Reagents

All reagents were purchased and used as received unless otherwise indicated. Oregon Green® 488 Cadaverine, 5-isomer was obtained from Invitrogen (Waltham, MA). CY-09 was acquired from Tocris Bioscience (Bristol, UK) and resuspended in DMSO to make a 1 mM stock solution for experimental procedures. Rough strain lipopolysaccharides (LPS) from E. coli and nigericin sodium salt were purchased from Sigma-Aldrich (St. Louis, MO) and reconstituted to generate stock solutions of 1 mg/mL in PBS and 1 mM in ethanol, respectively. Rabbit anti-IBA1 antibody (Catalog No. ab178847) was obtained from Abcam (Cambridge, UK) and Alexa Fluor® 594-conjugated anti-rabbit secondary antibody (Catalog No. A32754) was purchased from Invitrogen (Waltham, MA). DyLight® 649-conjugated GSL I Isolectin B4 (IB4) was acquired from Vector Laboratories (Burlingame, CA). HPLC grade solvents were obtained from Fisher Scientific (Waltham, MA). All other reagents, including deuterated solvents, were purchased from Sigma-Aldrich (Milwaukee, WI).

Chemical synthesis and characterization

General techniques

Moisture-sensitive reactions were performed in oven-dried glassware under a positive pressure of nitrogen or argon. Air and moisture-sensitive compounds were introduced via syringe or cannula through a rubber septum.

High-performance liquid chromatography (HPLC)

The analytical HPLC was performed on a Waters™ reverse-phase HPLC system equipped with a fluorescence detector set to $\lambda_{ex}/\lambda_{em}$ = 510/540 nm (Waters Corporation, Milford, MA). Chromatographic separation was carried out on 40 µL of injected sample using a Kinetex C18 reverse-phase column (250 x 4.6 mm, 5 µm particle size, and 100 Å pore size; Phenomenex®, Torrance, CA) maintained at 42 °C, a mobile phase consisting of Solvent A (H_2O, 2% Solvent B and 0.2% TFA) and Solvent B (MeOH:ACN:IPA, 1:1:1, and 0.2% TFA), and a flow rate of 0.6 mL/min. The following gradient was used: 0 to 2 min, 15% B (=15% Solvent B and 85% Solvent A); 2 to 10

min, linear increase from 15% to 97% B; 10 to 20 min, 97% B. The chromatogram was acquired and processed using Empower® 3 software (Waters Corporation, Milford, MA).

Spectroscopic analysis

The excitation and emission spectra were obtained using a Cytation 5 microplate reader (BioTek Instruments, Inc., Winooski, VT). High-resolution mass spectrometry (HRMS) was performed using an LTQ Orbitrap XL™ hybrid FT mass spectrometer (Thermo Scientific, Waltham, MA). The ^{1}H-NMR spectrum was collected on a Bruker Ultrashield™ 400 MHz spectrometer (Billerica, MA). The data were processed using squared sinebell window function, symmetrized, and displayed in magnitude mode. Chemical shifts were reported in ppm using the residual of MeOD as the internal standard. The following abbreviations were used: s = singlet, d = doublet, br = broad, t = triplet, q = quartet and m = multiplet.

Synthesis of InflammaProbe-1

InflammaProbe-1 was synthesized by adding CY-09 (11.8 µmol), 1-hydroxybenzotriazole hydrate (HOBt; 11.8 µmol), N, N- diisopropylethylamine (DIPEA; 11.8 µmol), and 1-(3-dimethylaminopropyl)-3-ethylcarbodiimide hydrochloride (EDCl; 11.8 µmol) to a stirred solution of Oregon Green® 488 Cadaverine, 5-isomer (10.07 µmol) in dimethyl formamide (DMF; 2 mL) at 25 °C. The resultant mixture was stirred for 2 days at 25 °C. Removal of the solvent in vacuo afforded a residue that was purified by silica gel column chromatography using $CHCl_3$:MeOH:NH_4OH (35:7:1) to give an orange solid. Yield = 31%. InflammaProbe-1 was dissolved in DMSO to a 1 mM stock solution for experimental procedures, unless otherwise indicated. The synthesis scheme shown in Figure 1b was created with ChemDraw Professional (V20.1.1; PerkinElmer Informatics, Inc., Waltham, MA).

Characterization of InflammaProbe-1

Excitation and emission spectral scan (in EtOH containing 10% DMSO), $\lambda_{Ex}/\lambda_{Em}$ = 510/540 nm (Figure 1b). HPLC (λ_{Ex}= 510 nm), t_R= 14.15 min, 95% purity (Figure S1). HRMS (ESI+): m/z $[M+H]^+$ calcd for $C_{45}H_{32}F_5N_3O_8S_2$, 902.1624; Found, 902.1647 (Figure S2). ^{1}H-NMR (400 MHz,

MeOD): δ 8.79 (br,1H), 8.45 (br,1H), 8.22-8.17 (m,1H), 8.08 (m, 2H), 7.95 (m, 1H), 7.78 (m, 2H), 7.69 (m, 2H), 7.61 (m, 2H), 7.58 (m, 1H), 7.48 (m, 1H), 7.40 (m, 1H), 7.36 (m, 1H), 6.82 (d, J = 7.6 Hz, 1H), 6.39 (d, J = 11.2 Hz, 1H), 5.43 (s, 2H), 3.52 (m, 4H), 1.76 (m, 4H), 1.56 (m, 2H).

Animal feeding and housing conditions

C57BL/6 mice were group-housed in ventilated cages according to their randomly assigned experimental group, and were maintained under a 12 h:12 h light:dark cycle at 22 ± 2 °C in an institutional animal care facility. They were provided clean water (Nashville Metro Water Services, Nashville, TN) and a standard diet consisting of 4.5% fat (PicoLab® Rodent Diet 5L0D; LabDiet St. Louis, MO) ad libitum. Mice were humanely sacrificed by CO_2-induced asphyxiation followed by cervical dislocation.

Confocal microscopy and image processing

Confocal fluorescence microscopy was performed using an LSM 710 inverted microscope (Zeiss™, Jena, Germany). Image acquisition was conducted using ZEN Black Edition (V2.4, SP1; Zeiss™, Jena, Germany). Images were processed uniformly and identically across control and experimental groups using ZEN Blue edition (V2.6; Zeiss™, Jena, Germany) and PowerPoint (V2112; Microsoft, Redmond, WA). Refer to Tables S1-4 for details on microscope configurations and image processing steps. Microscopy experiments and image processing were conducted in accordance with recommendations for rigor and reproducibility established in the literature.[1-4]

Immunostaining of choroidal tissue for ex vivo imaging

After in vivo imaging, all LCNV mice were sacrificed. Their eyes were enucleated and fixed in 10% neutral buffered formalin (NBF) overnight at 4 °C. The following morning, the eyes were washed with PBS and kept in PBS at 4 °C for two days. Then, the choroids were dissected, washed with PBS, and blocked/permeabilized in a solution containing wash buffer (TBS, 0.05% sodium azide, 0.33% Tween 20, and 0.0033% Triton-X), 1% bovine serum albumin (BSA), 10% donkey serum, and 0.2% fish gelatin for 2 h at room temperature. They were then exposed to a solution containing wash buffer, 0.2% BSA, and anti-IBA1 antibody (1:500 dilution) and left on a shaker overnight at 4 °C. The next morning, the choroids were washed twice in wash buffer for two minutes each time,

exposed to a solution containing wash buffer, 0.2% BSA, DyLight® 649-conjugated IB4 (1:100 dilution) and Alexa Fluor® 594-conjugated anti-Rabbit antibody (1:100 dilution), and placed on a shaker for 2 h at room temperature. Then, they were washed twice with wash buffer for two minutes each time and mounted on microscope slides with Prolong™ Diamond Antifade Mountant with DAPI (Invitrogen, Waltham, MA) in preparation for imaging.

Anesthesia and pupillary dilation

Mice were anesthetized with a 70 µL IP injection of a 1:1:2 mixture of Ketamine (85.7 mg/kg; Hospira, Inc., Lake Forest, IL), Xylazine (17.9 mg/kg; Akorn, Inc., Lake Forest, IL), and 0.9% saline. Pupillary dilation was achieved with a drop each of 0.5% tropicamide, (Sandoz, Basel, Switzerland) and 2.5% phenylephrine (Paragon BioTeck, Inc., Portland, OR). The corneas were numbed with a drop of 0.5% proparacaine (Akorn, Inc., Lake Forest, IL).

Electroretinography setup

After anesthetization and pupillary dilation, mice were placed on a warm stage to maintain physiological body temperature. Next, a circular gold electrode was placed around each cornea, a reference electrode was inserted subcutaneously between the eyes, and a ground electrode was inserted subcutaneously at the base of the tail. Before starting the ERG measurements, two drops of 0.9% saline were placed on each eye to maintain hydration and improve electrical conductivity.

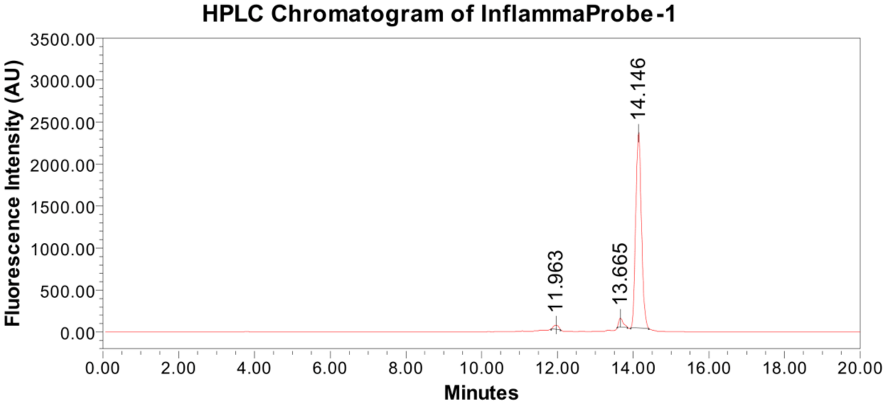

Peak Analysis
Processed Channel: 2475ChA ex510/em540

	Processed Channel	Retention Time (min)	% Area
1	2475ChA ex510/em540	11.963	1.73
2	2475ChA ex510/em540	13.665	3.19
3	2475ChA ex510/em540	14.146	95.08

Figure S1. HPLC chromatogram of InflammaProbe-1.

The InflammaProbe-1 that was used for biological assays was more than 95% pure based on a reversed phase HPLC monitored at 510 nm.

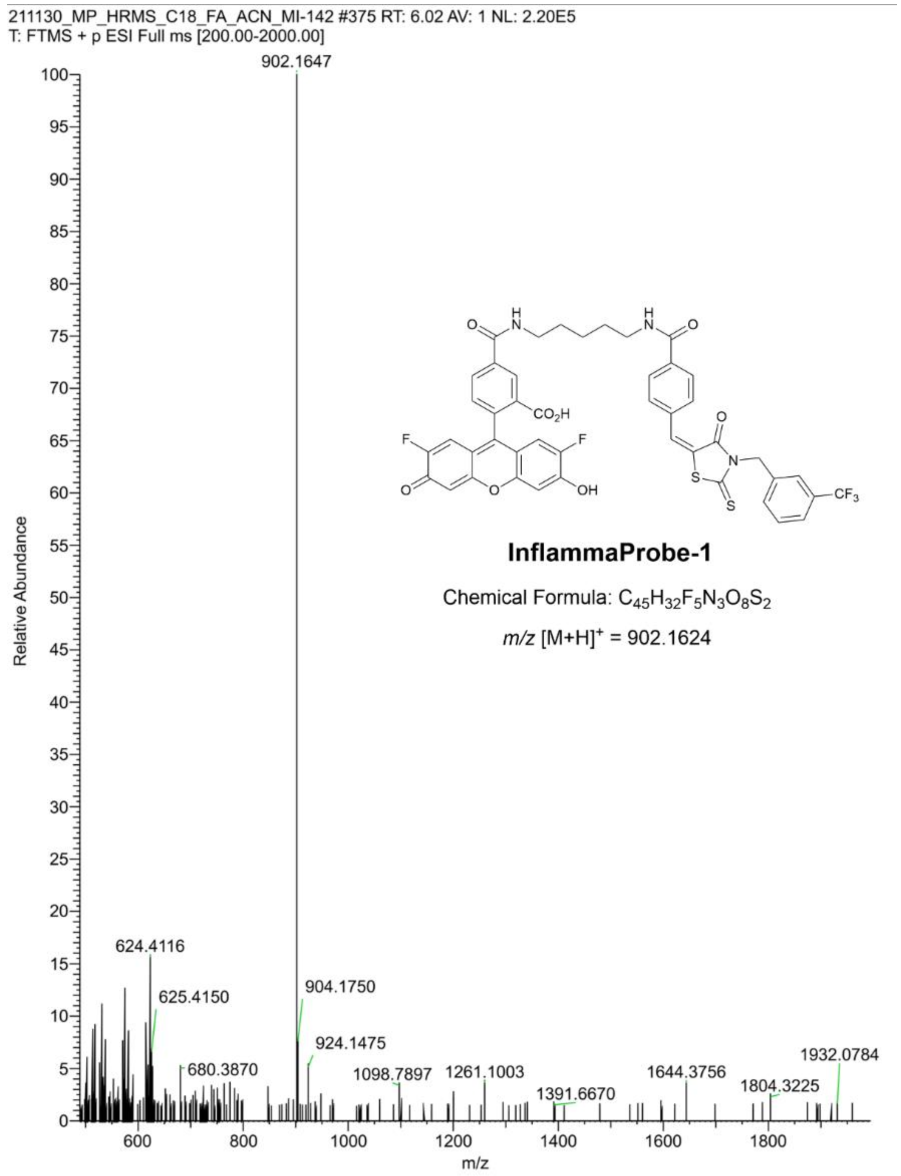

Figure S2. HRMS spectrum of InflammaProbe-1.

HRMS (ESI+): m/z [M+H]+ calcd for $C_{45}H_{32}F_5N_3O_8S_2$, 902.1624; Found, 902.1647.

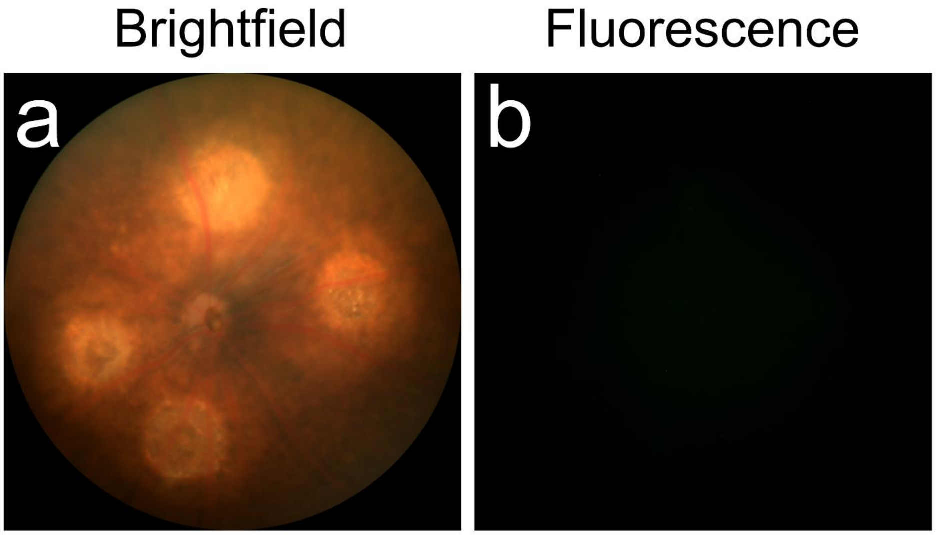

Figure S3. In vivo retinal imaging of Oregon Green® 488 dye control.
(a) Brightfield and **(b)** fluorescence fundus images of mouse laser-induced choroidal neovascularization (LCNV) taken 6 h after a 5.46 mg/kg intraperitoneal injection of Oregon Green® 488 cadaverine, 5-isomer on day 4 post LCNV. No detectable fluorescence was observed.

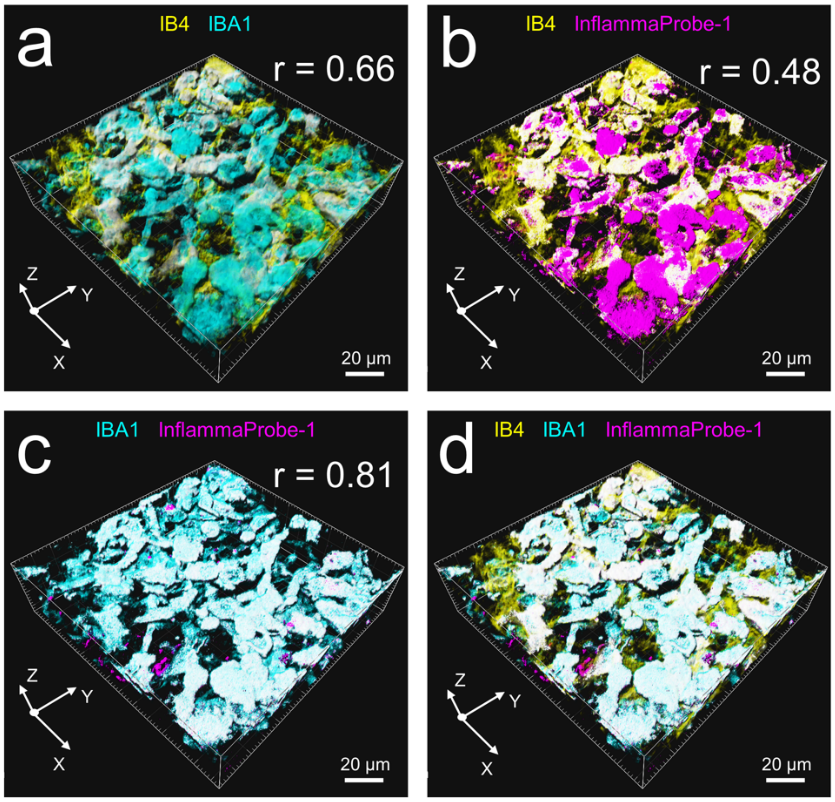

Figure S4. Degree of colocalization of InflammaProbe-1 fluorescence with IBA1 and IB4 in LCNV lesions.

Three-dimensional reconstruction of the stained LCNV lesion using Z-stacked confocal fluorescence images at 63x magnification. Areas of overlap between at least two stains appear white. The degree of correlation within each pair of stains is indicated by Pearson's correlation coefficient (r). The high degree of correlation between InflammaProbe-1 and IBA1, relative to that of InflammaProbe-1 and IB4, suggests that InflammaProbe-1 targets not endothelial cells, but NLRP3-associated macrophages.

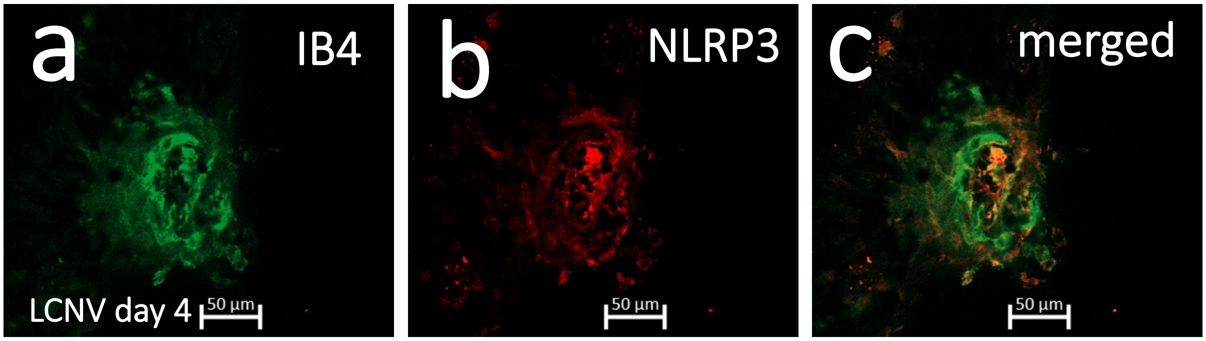

Figure S5. *Ex vivo* validation of CNV lesion associated NLRP3 expression in mouse LCNV.

Localization of NLRP3 inflammasomes, an inflammatory biomarker expressed in LCNV lesions of adult C57BL/6 mice. (a) Fluorescence dye conjugated IB4 was used as a counter stain to visualize choroidal neovascular lesions in LCNV. (b) NLRP3 expression was localized in LCNV lesions using an antibody specific for NLRP3. Adult mice were sacrificed on day-4 post-laser injury, and choroidal tissues were dissected and flatmounted for fluorescence confocal imaging. Non-lasered choroidal tissue areas showed no positive staining for NLRP3, suggesting that NLRP3 is associated with injured LCNV lesions.

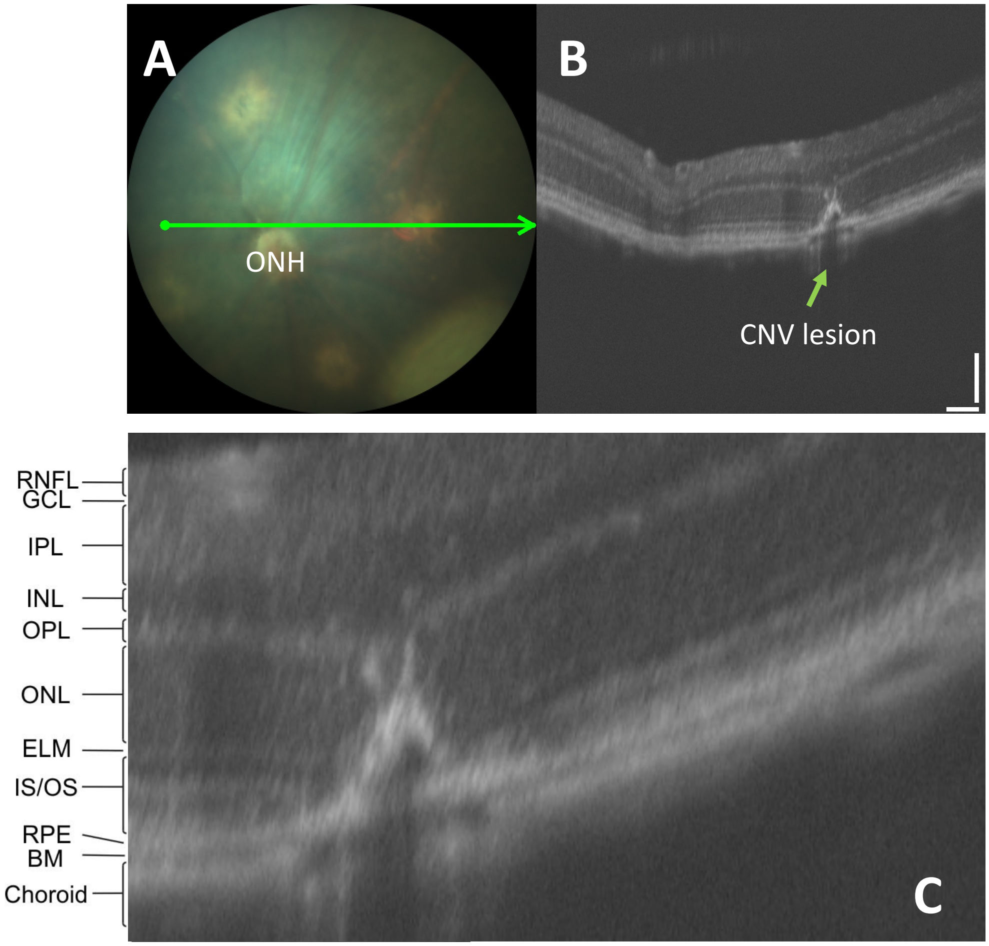

Figure S6. Validation of CNV lesion to confirm rupture of the Bruch's membrane using OCT imaging of mouse LCNV.

The green line in the OCT fundus image in **A** represents the location of the b-scans in the corresponding *en face* image in **B**. (**C**) Rupture of the Bruch's membrane was confirmed in LCNV lesions. Abbreviations: RNFL = retinal nerve fiber layer, GCL = ganglion cell layer, IPL = inner plexiform layer, INL = inner nuclear layer, OPL = outer plexiform layer, ONL = outer nuclear layer, ELM = external limiting membrane, IS/OS = photoreceptor inner segment/outer segment, RPE = retinal pigment epithelium, BM = Bruch's membrane.

Table S1. Microscope configuration for in vitro imaging at 63x.
Microscopy and image processing details pertaining to Figure 3a-i.

**Image Dimensions**		
Scaling (per pixel)	0.26 µm x 0.26 µm	
Image size (pixels)	512 x 512	
Image size (scaled)	134.95 µm x 134.95 µm	
Bit depth	8 bit	
**Acquisition Information**		
Software	ZEN Black Edition (V2.4, SP1)	
Microscope	Zeiss™ LSM 710, Inverted, AxioObserver	
Objective	Plan-Apochromat 63x/1.40 Oil DIC M27	
Beam splitter	MBS 458/514	
Acquisition mode	Sequential multichannel acquisition	
	**DAPI**	**InflammaProbe-1**
Excitation wavelength/laser	405 nm: 2.0%	514 nm: 4.0%
Emission wavelength	462 nm	537 nm
Detector type	PMT	PMT
Detector gain	650	699
**Image Processing**		
**Software**	**Processing steps**	
ZEN Blue edition (V2.6)	Pseudocolor, scale bar, and merge	
PowerPoint (V2112)	Brightness: +20%	

Table S2. Microscope configuration for in vitro imaging at 20x.
Microscopy and image analysis details pertaining to Figure 3j.

**Image Dimensions**		
Scaling (per pixel)	0.83 µm x 0.83 µm	
Image size (pixels)	512 x 512	
Image size (scaled)	425.10 µm x 425.10 µm	
Bit depth	8 bit	
**Acquisition Information**		
Software	ZEN Black Edition (V2.4, SP1)	
Microscope	Zeiss™ LSM 710, Inverted, AxioObserver	
Objective	Plan-Apochromat 20x/0.8 M27	
Beam splitter	MBS 458/514	
Acquisition mode	Sequential multichannel acquisition	
	**DAPI**	**InflammaProbe-1**
Excitation wavelength/laser	405 nm: 2.0%	514 nm: 2.0%
Emission wavelength	462 nm	537 nm
Detector type	PMT	PMT
Detector gain	650	699
**Image Analysis**		
**Software**	**Analysis**	
Fiji ImageJ (1.53q)	Calculation of raw integrated density	

Table S3. Microscope configuration for ex vivo imaging at 10x.

Microscopy and image processing details pertaining to Figure 5a-d.

**Image Dimensions**			
Scaling (per pixel)	1.66 µm x 1.66 µm		
Image size (pixels)	512 x 512		
Image size (scaled)	850.19 µm x 850.19 µm		
Bit depth	8 bit		
**Acquisition Information**			
Software	ZEN Black Edition (V2.4, SP1)		
Microscope	Zeiss™ LSM 710, Inverted, AxioObserver		
Objective	Fluar 10x/0.50 M27		
Beam splitter	MBS 633/561/488		
Acquisition mode	Sequential multichannel acquisition		
	**IB4**	**IBA1**	**InflammaProbe-1**
Excitation wavelength/laser	633 nm: 2.0%	561 nm: 2.0%	514 nm: 2.0%
Emission wavelength	697 nm	579 nm	563
Detector type	PMT	PMT	PMT
Detector gain	741	583	822.9
**Image Processing**			
**Software**	**Processing steps**		
ZEN Blue edition (V2.6)	Pseudocolor, scale bar, merge		
PowerPoint (V2112)	annotations		

Table S4. Microscope configuration for ex vivo imaging at 63x.
Microscopy and image processing details pertaining to Figure 5e-h.

**Image Dimensions**			
Scaling (per pixel)	0.26 µm x 0.26 µm		
Image size (pixels)	512 x 512		
Image size (scaled)	134.95 µm x 134.95 µm		
Bit depth	8 bit		
**Acquisition Information**			
Software	ZEN Black Edition (V2.4, SP1)		
Microscope	Zeiss™ LSM 710, Inverted, AxioObserver		
Objective	Plan-Apochromat 63x/1.40 Oil DIC M27		
Beam splitter	MBS 633/561/488		
Acquisition mode	Sequential multichannel acquisition		
	**IB4**	**IBA1**	**InflammaProbe-1**
Excitation wavelength/laser	633 nm: 2.0%	561 nm: 2.0%	514 nm: 2.0%
Emission wavelength	697 nm	579 nm	563
Detector type	PMT	PMT	PMT
Detector gain	524.2	517.3	787.8
**Image Processing**			
**Software**	**Processing steps**		
ZEN Blue edition (V2.6)	Pseudocolor, scale bar, merge		
PowerPoint (V2112)	annotations, arrows		

SUPPLEMENTARY REFERENCES

1. Lee, J. Y.; Kitaoka, M., A beginner's guide to rigor and reproducibility in fluorescence imaging experiments. *Mol Biol Cell* **2018,** *29* (13), 1519-1525.

2. Jost, A. P.; Waters, J. C., Designing a rigorous microscopy experiment: Validating methods and avoiding bias. *J Cell Biol* **2019,** *218* (5), 1452-1466.

3. Jambor, H.; Antonietti, A.; Alicea, B.; Audisio, T. L.; Auer, S.; Bhardwaj, V.; Burgess, S. J.; Ferling, I.; Gazda, M. A.; Hoeppner, L. H.; Ilangovan, V.; Lo, H.; Olson, M.; Mohamed, S. Y.; Sarabipour, S.; Varma, A.; Walavalkar, K.; Wissink, E. M.; Weissgerber, T. L., Creating clear and informative image-based figures for scientific publications. *PLoS Biol* **2021,** *19* (3), e3001161.

4. Schmied, C.; Jambor, H. K., Effective image visualization for publications - a workflow using open access tools and concepts. *F1000Res* **2020,** *9*, 1373.
